# The effects of bending on plasmonic modes in nanowires and planar structures

**DOI:** 10.1515/nanoph-2021-0449

**Published:** 2021-12-21

**Authors:** Edson P. Bellido, Isobel C. Bicket, Gianluigi A. Botton

**Affiliations:** Department of Materials Science and Engineering, McMaster University, Hamilton, Canada; Canadian Centre for Electron Microscopy, McMaster University, Hamilton, Canada; Canadian Light Source, Saskatoon, Canada

**Keywords:** antennas, coupling, EELS, hybridization, nanowires, plasmonics

## Abstract

In this work, we investigate the effects of bends on the surface plasmon resonances in nanowires (NWs) and isolated edges of planar structures using electron energy loss spectroscopy experiments and theoretical calculations. Previous work showed that the sharp bends in NWs do not affect their resonant modes. Here, we study previously overlooked effects and analyze systematically the evolution of resonant modes for several bending angles from 30° to 180°, showing that bending can have a significant effect on the plasmonic response of a nanostructure. In NWs, the modes can experience significant energy shifts that depend on the aspect ratio of the NW and can cause mode intersection and antinode bunching. We establish the relation between NW modes and edge modes and show that bending can even induce antinode splitting in edge modes. This work demonstrates that bends in plasmonic planar nanostructures can have a profound effect on their optical response and this must be accounted for in the design of optical devices.

## 1 Introduction

Nanowires (NWs) that support surface plasmon resonances can potentially be used as components in the realization of integrated photonic circuits with nanoscale elements due to the NW’s ability to confine light into sub-wavelength volumes [1–3]. The desire to shrink photonic circuits below the diffraction limit prompts the development of plasmonic equivalents of many photonic circuit components [3, 4], such as S-bends, Y-splitters, Mach–Zehnder interferometers, ring resonators, and so on [5–8], many of which rely on waveguide bending in order to function. Even in a simple waveguide, bending is known to cause losses in both photonic and plasmonic circuits [4, 9, 10]; these losses must be mitigated and the effects of bending must be thoroughly understood to enable the engineering of effective plasmonic circuits.

To understand the effects of bending on the confined modes supported by NWs, imaging techniques with high spatial resolution exceeding that of diffraction-limited optical techniques are required. Thus, electron probe techniques such as electron energy loss spectroscopy (EELS), performed inside a scanning transmission electron microscope (STEM), are necessary for this type of analysis. The combination of high spatial resolution, limited only by delocalization [11], and spectral resolutions in the 10 meV range [12–15] makes EELS a very powerful characterization technique for plasmonic structures including rods and wires [16, 17], gaps [18], cubes [19] and structures of various shapes [20–24], as well as the coupling of plasmonic structures [25–28].

Bending in NWs has been previously studied by optical techniques and by electron energy loss spectroscopy (EELS). The results are somewhat contradictory, while in some studies there are claims that the presence of kinks and bends does have an effect on the plasmonic response [10, 29, 30], other works claim that bends and kinks do not affect the overall plasmonic response [31–33]. Most of these measurements focus on the effects of bending on the plasmon radiative field amplitude and not on the effect of the profile of plasmon modes. Far-field optical measurements have demonstrated that bending in NWs produces amplitude losses that depend on the radius of curvature of a bend, the width of the nanowire, the excitation wavelength [10], and the polarization of the incident light [29]. Also, in cases where single crystal NWs are sharply bent, the bending can induce cracks and defects that can act as scattering points, although the losses due to these scattering points are small [31]. Near-field optical measurements have shown that bending losses on the evanescent field of NW are small compared to propagation losses [30]. Also, using photo-bleaching to characterize the near-field of NWs made of nanoparticle chains, plasmon propagation around 90° corners was shown [33]. The effects of bending on the plasmon modes of NWs have been studied by our group before [32]. That work indicated that the presence of bends in NWs does not affect the plasmon modes. However, the bending angles in the NWs investigated in that particular work were large and the NWs were very long, relative to their thickness (*i.e.*, with high aspect ratio). In this work, we analyze the evolution of plasmon modes in NWs more systematically, studying the effects of bending angles methodically changing from 180° (*i.e.*, corresponding to a straight NW) to 30° (a NW with a sharp kink). Following a similar systematic approach, we also analyze the bending effect on the edge modes of planar nanostructures.

## 2 Materials and methods

The one-dimensional longitudinal plasmon modes in a NW can be described by their number of nodes, *m*. As previously shown, NWs can support high order modes depending on their length. To study the effect of bending on these modes, we analyzed the evolution of the NW modes for several bending angles. For this analysis, two sets of silver NWs with a rectangular cross-section and with bending angles from 30° to 180° were fabricated by electron beam lithography on 50 nm thick silicon nitride membranes for transmission electron microscopy. The silicon nitride membranes are fabricated and cleaned in order to avoid sulfidation of the silver NWs, by Norcada Inc. Also, to avoid degradation of the NWs, experiments are carried out soon after fabrication and samples are then stored under vacuum. One set of wires was 2 μm long, 30 nm thick, and 70 nm wide (28.6 aspect ratio), and another set of wires was 850 nm long, 30 nm thick, and 55 nm wide (15.5 aspect ratio). The EELS spectra were acquired in a monochromated STEM FEI Titan operated at 80 keV with the monochromator under accelerating conditions with a potential of 800 V. The data was post-processed by applying Richardson–Lucy deconvolution [14] to enhance the effective energy resolution of the measurement and visualize the fine features in greater detail.

To further support the experimental results, we performed numerical simulations of a larger range of bent NWs using the MNPBEM Matlab toolbox [34, 35], which applies the boundary element method to solve the Maxwell equations. The silver NWs were simulated using a tabulated experimental dielectric function [36], and, to account for the effect of the silicon nitride substrate and vacuum on the NWs, the surrounding environment was modeled using an effective dielectric function of 3.5. All calculations except for the eigenmode calculations were done using the retarded approximation. Eigenmode calculations were performed to calculate the quasistatic normal modes of the system. Electric fields for the eigenmodes and structures under electron beam excitation were calculated in a plane 10 nm below the surface of the NW. Differences in the energy between the spectra and eigenenergies are a result of differences between the retarded BEM calculation and the quasistatic eigenmode solver of MNPBEM.

## 3 Results and discussion

### 3.1 Energy shifts and antinode bunching

**Figure 1:**
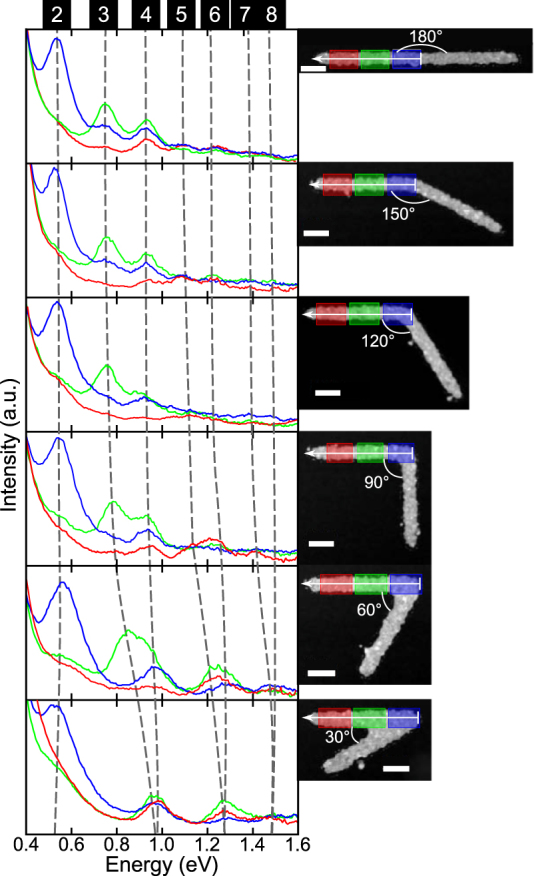
Evolution of NWs’ EEL spectra as a function of bending angle (left) acquired and averaged over three color-coded locations shown in the annular dark field (ADF) images (right). The scale bar in all ADF images is 100 nm.

Figure 1 shows EELS spectra acquired at three distinct locations on 850 nm long isolated NWs, indicated in the annular dark-field (ADF) images, for NWs with different bending angles. In the straight wire, we can identify up to seven peaks that correspond to resonant modes with *m* = 2 to 8. When we decrease the bending angle from 180° to 90° the changes in the resonant energy of the modes are not significant. For the 60° and 30° angles, the modes *m* = 3, 5, and 7 experience a significant blue shift, with changes in energy up to 180 meV between 90° and 30°. Due to this blue shift, modes 3 and 4 intersect in energy when the bending angle is 30°. Similarly, modes 5 and 6 intersect in energy in the NW with 30° bending angle. Modes 7 and 8 cannot be distinguished in all the NWs due to their smaller excitation probability compared with lower order modes, and due to roughness in the NWs’ surface. However, we can observe that the energy shift of mode 7 causes the energy intersection of modes 7 and 8 at a 60° bending angle. In Figure S1 in the supplementary information document, we observe a similar behavior, this time in the 2 μm long NWs. Small energy shifts for bending angles above 90° and a significant shift for smaller angles can be observed. However, for the 2 μm long NW only modes *m* ≥ 5 experience the same energy intersection observed in the 850 nm NWs while modes *m* = 3 and 4 do not. This result indicates that the effects of bending in NWs depend on the dimensions of the NWs, as we will discuss further below.

**Figure 2:**
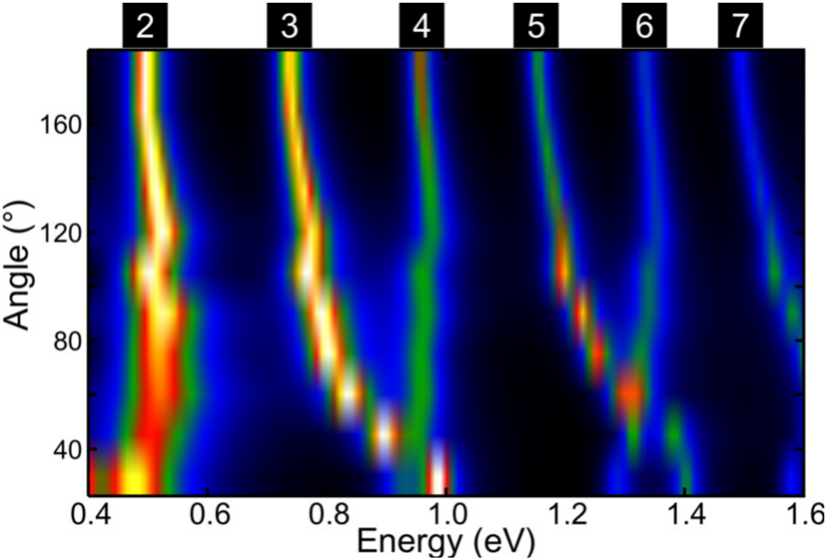
Simulated EEL spectra as a function of bending angle of the 15.5 aspect ratio silver NWs. The spectra show the energy shifts on the resonant modes that, at low bending angles, cause even and odd modes to intersect in energy.

The simulated spectra in Figure 2 confirm our experimental results. We observe the same trend found in the experimental spectra, with modes 3–4, and 5–6 intersecting in energy at small bending angles. However, a different phenomenon is observed in the simulation of modes 2, 4, and 6, where we observe initially a small blue shift of the resonant peaks, followed by a red shift at small angles. The changes in energy for modes 2, 4, and 6 from 180° to 30° are small enough that we cannot accurately separate them experimentally from small energy changes due to small imperfections and differences between NWs caused by the lithography process. In the simulations, we also observe that the intersection in energy for modes 3–4, and 5–6 occurs at 40° and 55°, respectively. The energy intersection for each pair of modes at different bending angles indicates that each mode experiences the effects of the bend to a different extent, with higher order modes being significantly more affected than their lower order counterparts. For the dipolar mode (*m* = 1) we did not detect any significant changes for the range of angles and NWs analyzed. The simulations of the 2 μm long NWs, in Figure S2, also show a similar trend. In modes 2 and 4 we notice that there is only a blue shift at high angles, while only in mode 4 we observe that at 45° angle the mode stops blue shifting. These results indicate that, although there is a very small shift for angles above 90°, the change is negligible and very difficult to detect experimentally with current energy resolutions. This explains why in the work of Rossouw et al. no change in the modes was reported [32]. However, for small bending angles, depending on the NW geometry, the energy shift is no longer negligible, particularly for high order modes where NW modes even intersect.

**Figure 3:**
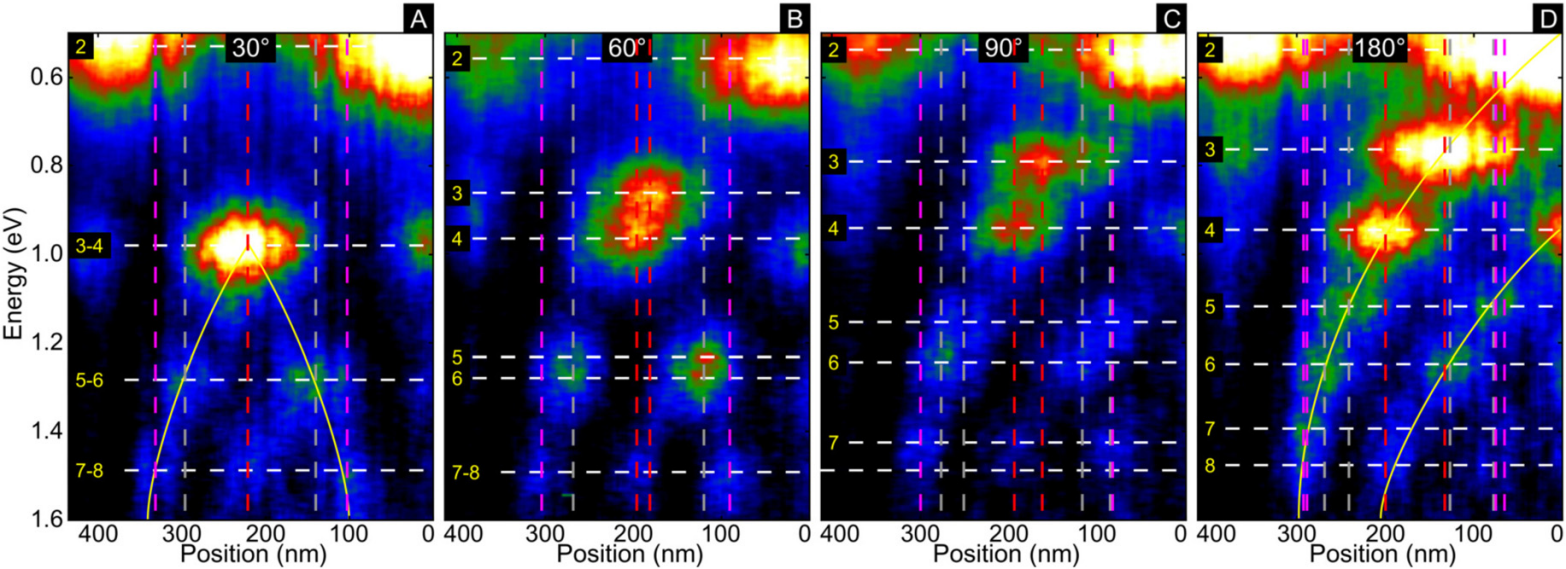
EELS intensity profiles of NWs with 30° (A), 60° (B), and 90° (C) bending angles, as well as of a straight NW (D). The figure shows how the nodal distribution of the NW modes changes due to the presence of a bend, inducing antinode bunching in energy and position. The EELS profiles are acquired over half of the NWs across their width in the regions indicated by the arrows in the ADF images in Figure 1. The vertical and horizontal lines are a guide to the eye of the center of the antinodes.

After discussing the energy shifts produced by the bending in NWs, we analyze now how the nodal distribution of the modes is affected by the presence of a bend. This concept is relevant to understand how light could be optimally coupled to NWs and how this effect can alter the distribution of their light emission. Figure 3 shows the experimental EELS intensity profile of a straight NW and of NWs with bending angles from 90° to 30°, acquired along the NW and integrated across the width of the NW in the region indicated by the white line with an arrow in the ADF images in Figure 1. It is important to highlight that the region of signal extraction is half the length of the NW and, due to symmetry, the other half of the NW has the same EELS profile. From Figure 3D, we observe the typical nodal distribution of a straight nanowire with antinodes moving away from the center of the nanowire as the mode order and energy increase [37]. This typical nodal distribution changes considerably for the 30° bent nanowire, with the modes 3–4, 5–6, and 7–8 merging and becoming degenerate as their antinodes cluster due to the presence of the bend in the middle of the NW. As shown in Figure 3, when the bending angle changes from 180° to 30° the center-to-center distance between the antinodes of modes 3 and 4 is reduced until the antinodes finally merge at 30°. The same effect is observed in modes 5 and 6. For example, in modes 3 and 4 the center-to-center distance between antinodes, initially 65 nm in the 180° NW, is reduced to 30 and 15 nm for 90° and 60°, respectively, to finally become only one antinode in the 30° NW. The same antinode bunching is observed in the 2 μm NWs in Figure S3. For the longer NW, even in the case of modes that do not intersect in energy at 30° (modes 3 and 4), the antinode distance between the modes is reduced from 36 nm in the 180° NW to only 10 nm in the 30° nanowire. These results are a clear indication of antinode bunching produced by the presence of a bend in the NWs. Because of this antinode bunching, the nodal distribution of the edge modes in the 30° NW, shown in Figure 3A, resembles more the distribution of a nanowire half the size of the actual nanowire. Intuitively, these new results can be somewhat expected if we realize that, in the limit of a bending angle approaching 0°, we have a nanowire that is half the size of the original nanowire. However, to explain why the modes intersect at angles larger than 0° and why the angles are different for each pair of modes we need to analyze the evolution of eigenmodes in the NWs.

### 3.2 NW aspect ratio and eigenmode analysis

**Figure 4:**
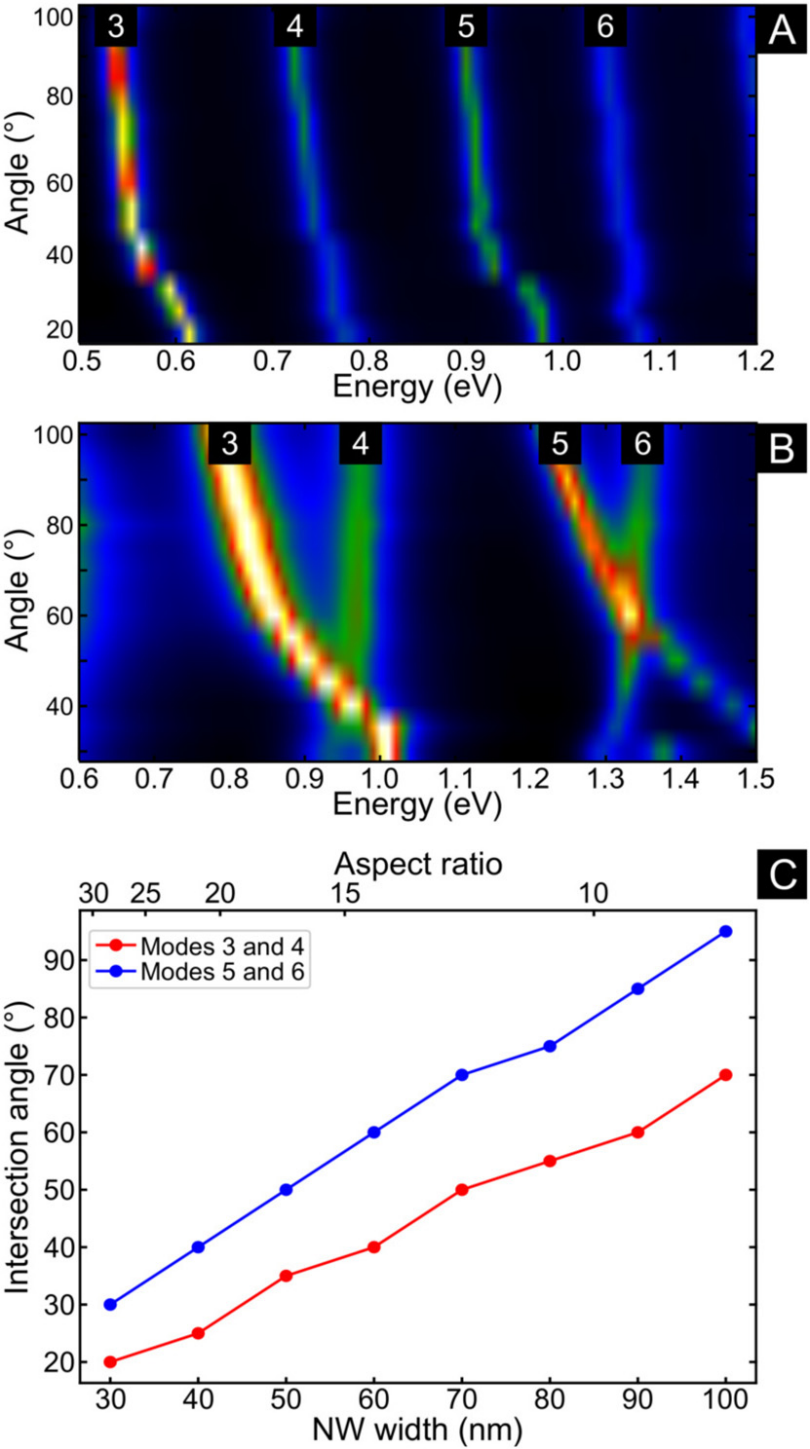
Simulated EEL spectra as a function of bending angle for NWs 850 nm long and with 20 nm (A), and 60 nm (B) width (42.5 and 14.2 aspect ratio, respectively). (C) shows how the intersection angle changes as a function of NW width and aspect ratio for modes 3–4 and 5–6.

To better understand how the effects of bending evolve as we change the aspect ratio of the nanowire, we simulated multiple NWs with the same length (850 nm) but with different widths and bending angles. Figures 2 and S2 show the EEL spectra as a function of bending angle for NWs with aspect ratios of 15.5 and 28.6, respectively. Also, Figure 4A and B shows the EEL spectra for NWs with 20 and 60 nm width (42.5 and 14.2 aspect ratio, respectively) as a function of bending angle. This figure is complemented by surface charge and electric field calculations for various bend angles and energies, presented in Figures S5–S7. The plasmonic response due to bending for these NWs for the range of investigated angles is different. For the high aspect ratio NW (*i.e.*, 42.5), the modes do not intersect in energy and only a small blue shift rate is observed. In contrast, for lower aspect ratio NWs, we observe that the modes intersect, or cross, in energy and their behavior is similar to the NWs we studied experimentally. Therefore, the aspect ratio of the NWs has a strong influence on the effects of bending. For NWs with higher aspect ratios, the effects of bending are reduced and we do not observe an energy crossing for the NW modes, even at bending angles as small as 20°. In addition, we observe that the odd and even modes are able to couple more efficiently across the gap under electron beam excitation of high aspect ratio NWs (Figures S5–S7).

This dependence on the aspect ratio also explains why Rossouw et al. did not observe any change in the modes for the bent NWs they studied [32], since the aspect ratios of their NWs were higher than 42.5. In Figure 4C we observe that, as the width of the NW decreases (*i.e.*, when the aspect ratio decreases), the angle at which the energy crossing occurs decreases. Therefore, for NWs with aspect ratios higher than 42.5 the energy crossing will happen below 20°. Intuitively, we can understand this phenomenon if we consider that high aspect ratio NWs support more confined surface plasmon modes that are able to bend through smaller bending angles. These results suggest that we can control the effects of bending by changing the aspect ratio of NWs. This effect is particularly significant for waveguiding applications, in which it is necessary to understand and control the effects of bending. For example, in applications where bending would reduce the efficiency of a device, the impact of bending effects can be considerably reduced by decreasing the width of the NWs, while still keeping the topology of the waveguide. For short wires, however, this would impose an ultimate nanoscale limit as wire thicknesses cannot be fabricated with ever-decreasing dimensions.

**Figure 5:**
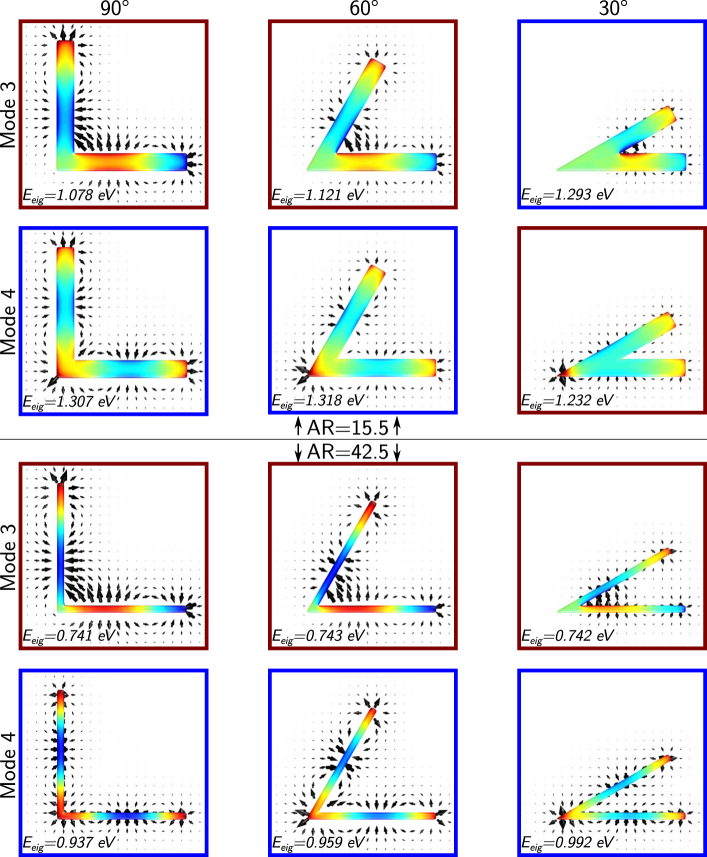
Evolution of eigenmodes 3 and 4 showing the changes in eigenenergy, charge distributions, and electric field distributions of the modes at different bending angles for NWs with aspect ratios of 15.5 (top two rows) and 42.5 (bottom two rows). The red boxes indicate the lower energy mode and the blue boxes the higher energy mode, highlighting the energy crossing of modes 3 and 4 of the NW with 15.5 aspect ratio.

In order to understand the fundamental reasons generating the bending effect in NWs, we investigated the eigenmodes of the bent NWs. The evolution of eigenmodes 3 and 4 for selected bending angles and NWs is shown in Figure 5 for NWs with low aspect ratio (15.5, shown on the top panel) and high aspect ratio (42.5, shown on the bottom panel). The evolution of these eigenmodes provides exquisite insight into the self-interaction of the plasmon resonant modes within a bent NW and the reason for the observed energy shifts. The self-interactions in plasmonic structures refer to the eigencharge-mediated interactions [38–40], which are different from the field-mediated electrostatic interactions between multiple plasmonic structures [41–43]. Both types of interactions can generate mode coupling and the observation of anticrossing, however, their energy response is reversed. While in the field-mediated case, the interaction of charges of the opposite sign induces a red shift, in the self-interaction case, it produces a blue shift because to support a mode it is necessary to maintain the polarization of the charges within the structure and more energy is required to oppose the attraction of charges of equal sign. Similarly, in the interaction of charges of equal signs in a field-mediated interaction, a blue shift is induced instead of the red shift induced in the self-interaction case. As discussed above, for large bending angles all the modes experience a small blue shift. From the eigenmode distributions we observe that, at large angles (*i.e.*, 90°, 120°, 180° in eigenmodes 3 and 4 in Figures 5 and S8), the antinode distribution only experiences a small perturbation, this perturbation being smaller for high aspect ratio NWs. This perturbation is responsible for the blue shift observed in the experiments and simulations, and arises from the eigenmode charge interactions within a structure. If antinodes of opposite charges are closer due to the presence of a bend, more energy will be required to maintain the polarization within an NW, thus the mode energy increases. For this reason, for large bending angles (*i.e.*, above 90° for NWs with high aspect ratio) we observe a blue shift as we reduce the bending angle due to the higher energy required to maintain polarization within the NW.

As we continue reducing the bending angle and enter the small angle regime (*i.e.*, below 90° for NW with low aspect ratio), the interaction between the antinodes closer to the bend becomes stronger. Geometrically, the relative area occupied by the bend itself increases and the length of the inner edge decreases as the aspect ratio decreases. In the low aspect ratio NWs, the charge distribution of eigenmode 3 shows the confinement of antinodes that have opposite charges and strong electric fields near the inside of the bend. Thus, the interaction between antinodes caused by this close proximity produces the rapid blue shift shown in Figures 1 and 2. The inner corner of the bend must support a charge node, and the geometrically larger bend of the low aspect ratio NW forces the mode 3 antinodes into a smaller volume on the inner edge, pushing the mode energy higher in lower aspect ratio structures.

In eigenmode 4 we observe that, as the bending angle decreases, the central antinode becomes confined on the bend and pushed to the outer edge of the wire, and the adjacent antinodes merge and start to interact within the NW and localize on opposite edges of the NW, supporting minimal electric fields inside the bend. The confinement of the central antinode and the interaction of the antinodes of equal charge reduce the field strength required to maintain the polarization across the bend of the NW, producing the red shift observed in Figures 1 and 2, which ultimately causes the crossing of even and odd modes. For NWs with a high aspect ratio, the rapid blue shift of the odd modes and the confinement of the central antinode of the even modes might only happen at very small bending angles. Contrary to the effects observed on the 15.5 aspect ratio NW, in the 42.5 aspect ratio NW, shown in the bottom set of NWs in Figure 5, we do not observe the strong confinement of charges in mode 3 nor the localization of the charge on the outer edge of the bend and the interaction of charges within the bend in mode 4. Based on our eigenmode calculations, it seems that in the case of the NW with 42.5 aspect ratio, eigenmode 5 maintains five charge antinodes with bend angles larger than 30°, and does not experience this rapid blue shift until much smaller bend angles. For higher order modes these eigenmode phenomena occur at even lower bending angles because the modes support a larger number of antinodes in the same physical space, therefore the confinement of the eigencharges is stronger. Thus, the larger the number of antinodes, the stronger the effect.

The effects of bending in a NW are also observed in the analysis of the surface charges and fields induced by the electron beam point excitation, as shown in Figures S5–S7 in the supplementary information document. In the simulations, the source of excitation is asymmetric, with the electron beam exciting surface plasmons from one side of the NW. These simulations show how the charge density of the surface plasmon propagates in the NW and around the bend. These simulations confirm our findings, showing that in NWs with a high aspect ratio the charge density distribution is weakly affected by the bend and more symmetrically distributed compared to low aspect ratio NWs. Similarly, lower-order modes are less affected by the bend than higher-order modes.

With the analysis of the eigenmodes in these NWs, we can now understand the origin of the energy shift caused by the bending and also the differences between NWs of different aspect ratios. We can also infer that the higher the mode order, the larger the number of antinodes close to the bend, and the stronger the effect that causes the mode energies to shift. As observed in Figures 4 and S2, the mode crossing occurs at larger angles for high order modes, indicating a stronger effect. It is also worth noting that the eigencharge interactions described in this work do not induce coupling that could give rise to the observation of anticrossing behavior, as described in previous works [38]. Instead, we observe the crossing of the modes because the interactions that produce the energy shifts in the bent NWs are between the charges of a single eigenmode and not between charges of orthogonal eigenmodes: one of the required characteristics for the formation of anticrossing and coupling within a plasmonic structure [40].

### 3.3 Bending on planar structures

**Figure 6:**
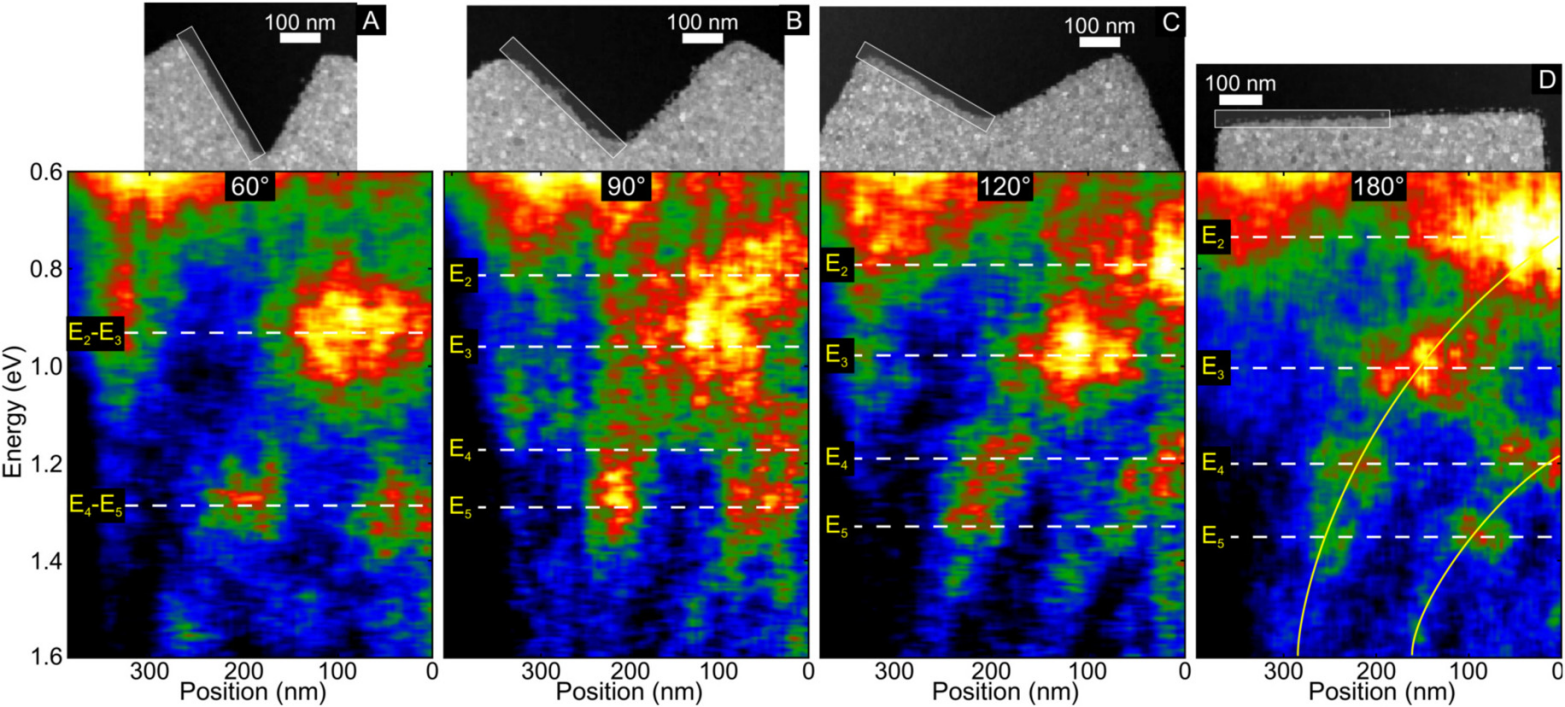
EELS intensity profile of isolated edges with 60° (A), 90° (B), and 120° (C) bending angles, as well as of a straight edge (D). It shows how the nodal distribution of the edge modes changes due to the presence of a bend. The EELS profiles are acquired in the region indicated by the boxes in the ADF images.

The effects of bending are not unique to NWs, however. Bends in the edges of planar structures also produce a similar remarkable effect on the plasmonic response of the structure, as was the case in NWs. Although the effects of bending in NWs and edges are technically different, they are, nonetheless, closely related. This relation can be understood if we review recently published work that showed that the localized surface plasmon resonances in planar nanostructures can be described by edge modes [39, 41, 44], [45], [46] and their coupling within the structure. In this context, we could arguably describe a NW as a planar nanostructure that supports edge modes. To confirm this description, in the supporting information document, we topologically transformed a NW into a nanosquare, which supports edge modes [45], by changing the width of the NW (Figure S9 in the supplementary information document). The results demonstrate that the NW modes are in fact bonding even edge modes formed by the strong coupling between the edges of the NW. Now that we have established the relationship between NW modes and edge modes we can study the effects of bending on edge modes. To analyze the effects of bending on edge modes, we need to isolate edges with bending angles from 180° (straight edge) to 30°. To do so, we fabricated the edges on a large silver slab, with 50 μm length from each corner of the confined edge as shown in the ADF images in Figure 6. The isolation of the edges, using such large structures, ensures that coupling with other edges of the structure is negligible.

Figure 6 shows the EELS intensity profile for half of the isolated edges for several bending angles, acquired over the region indicated by the box in the ADF images. On the straight edge, we observe the typical edge modes *E*_*n*_, with *n* = 2 to 5, with the antinodes moving away from the center of the edge. As we decrease the bending angle (*i.e.*, we make the angle more acute), we observe that the energy separation between modes *E*_2_–*E*_3_ and modes *E*_4_–*E*_5_ is reduced to ultimately overlap in energy for the edge with 60° bending angle. As in the case of the NWs, bending also produces antinode bunching. For the 90° angle, we observe that the central antinode of the even modes (*E*_2_ and *E*_4_) moves away from the center of the edge and splits into two antinodes with a node at the bend. For the 60° bending angle, in which even and odd modes intersect in energy, the antinodes of the modes merge, as we also observed in the NWs. Modes *E*_2_–*E*_3_ have an antinode at each side of the bend and modes *E*_4_–*E*_5_ have two antinodes at each side of the bend. Although we have established that edge and NW modes are related, the response to the presence of a bend is different. For the case of edge modes, modes *E*_2_–*E*_3_ and *E*_4_–*E*_5_ are the ones that intersect in energy, while in the NW modes 3–4 and 5–6 are the modes that intersect. This striking difference can be intuitively understood if we use the relationship we established above between NW and edge modes, and analyze the charge distribution of mode 4 of the NW, in Figure 5. In the case of the NWs, we noted that at small angles the reason for the red shift in even eigenmodes is due to the confinement of the central antinode over the bend in the external edge of the NW. In contrast, for the case of edge modes *E*_2_ and *E*_4_, the central antinode cannot be confined, since we do not have another edge as is the case in NWs, producing strong repulsion between each side of the edge close to the bend. This effect produces a significant blue shift (observed in the experimental results) which causes modes *E*_2_–*E*_3_ and *E*_4_–*E*_5_ to intersect in energy and become degenerate. For angles below 90°, the repulsion between the charges across the two edges around the bend is so strong that the central antinode is forced to split into two antinodes. The results show that edge modes are not significantly affected at large bending angles (above 120°). For smaller angles; however, the edge mode interaction caused by the bend can have a very strong effect on the plasmonic response of the structure. The formation of a node in the bend for small bending angles is similar to the formation of nodes in hollow or Babinet structures [47, 48], indicating the relation between edge modes and the modes supported by Babinet structures. The significance of this effect is profound, as structures become more complex and with a large number of features, such as kinks and corners as in the case of fractals structures, this effect could potentially limit the application of simple geometrical predictions based solely on feature size.

## 4 Conclusions

To summarize, we have used high spatial and energy resolution STEM-EELS to systematically analyze the effect of bending on NWs and edge modes in planar structures. We discovered that, under specific conditions, bending can have a significant effect on the plasmonic response of planar nanostructures. In NWs, the effect of bending depends on their aspect ratio, being stronger at small aspect ratios. These results settle the contradictory results found in the literature and show why, in some cases, the effects of bending in the optical response of NWs are so small that bending appears to have no effect at all. In NWs and edges, the bend causes the even and odd modes to shift in energy, and these shifts can cause mode intersection and antinode bunching. In edges, the effect can cause the central antinode to split into two antinodes forcing the formation of a central node. These effects will have to be considered in the realization of integrated plasmonic circuits that will use NWs as components. Also, the effects demonstrated here impose nanoscale limits in complex planar nanostructures, going beyond the traditional size considerations and highlighting the previously overlooked role of bends in the plasmonic response of nanostructures.

## Supplementary Material

See supplementary material for the detailed analysis of the 2 μm NWs, including EELS spectra and simulations, and the analysis of the relationship between NW modes and edge modes.

## Supplementary Material

Supplementary Material Details
