# Supplementary material for: The effects of bending on plasmonic modes in nanowires and planar structures
Source: Nanophotonics. 2021 Dec 21;11(2):305–14. doi: 10.1515/nanoph-2021-0449 (PMC9728462; doi:10.1515/nanoph-2021-0449)
Supplement: Supplementary file 1 — Supplementary Material Details [file j_nanoph-2021-0449_suppl.pdf]

Research Article - Supplementary Information

Edson P. Bellido, Isobel C. Bicket, and Gianluigi A. Botton*

The Effects of Bending on Plasmonic Modes in Nanowires and Planar Structures

https://doi.org/10.1515/sample-YYYY-XXXX
Received Month DD, YYYY; revised Month DD, YYYY; accepted Month DD, YYYY

Supplementary information

Figure S1 shows EELS spectra acquired at three distinct locations, indicated in the annular dark field (ADF) images, for 2 μm long nanowires (NWs) with a high aspect ratio of 28.6 and with different bending angles. In the straight wire, we can identify up to seven peaks that correspond to resonant modes with $m = 2$ to 8. When we decrease the bending angle from 180° to 90°, the changes in the resonant energy of the modes are not significant. For the 60° and 30° angles, the modes $m = 5, 7$ experience a blue shift. Due to this blue shift, modes 5 and 6 intersect in energy when the bending angle is 30°. Similarly, modes 7 and 8 intersect in energy in the NW with 30° bending angle. For these 2 μm long NWs, modes $m = 3$ and 4 do not experience the same energy intersection observed in the 850 nm NWs because the effects of bending in NWs depends on the aspect ratio of the NWs, as we discussed in the main text.

The simulations for the 2 μm NWs are shown in Figure S2. Because the fabricated structures are large (2 μm long), it is computationally expensive to simulate them, requiring a very large memory. For this reason, we simulate NWs at a scale five times smaller than the fabricated NWs. Despite the different scales of the experimental and simulated NWs, we observe a similar trend, with modes 5-6, and 7-8 intersecting at 30°. We also observe modes 9-10 intersecting at 45° bending angle. Mode 3 experiences a blue shift in two stages: a low linear shift rate of 0.1 meV/deg between 180° and 90°, and a faster shift rate of 0.83 meV/deg between 90° and 30°. The energy of modes 5, 7, and 9 blue shift with low rates of 0.1, 0.2, and 0.2 meV/deg respectively above 90°, however, their shift rate increases to an average of 1.3, 1.2, and 1.0 meV/deg respectively between 90° and 30°, which is more than five times the shift rate of angles above 90°. Similarly to the 850 nm NWs, modes 6, 8, and 10 first experience a small blue shift rate for high angles, then a red shift before converging with modes 5, 7, and 9 respectively. In modes 2 and 4 we notice that there is only a blue shift at high angles, and only in mode 4 we observe that, at 45°, the modes stop blue shifting. These results indicate that, although there is a very small shift for large angles, the change is negligible and very difficult to detect experimentally with current energy resolutions, explaining why previous work did not report any change in the modes that were analyzed.

In Figure S3A, we observe the typical nodal distribution of a straight NW with nodes moving away from the center of the NW as the mode order and energy increase. This typical nodal distribution changes considerably for the 30° bent NW (Figure S3A). We notice that the antinodes of modes 5 and 6 have clustered. Similarly, the antinodes of modes 7 and 8 clustered due to the presence of the kink at the middle of the NW. In modes 5 and 6, the antinodes, which are up to 28 nm apart in the 180° NW, shift to become only one antinode in the 30° NW. Similarly, in modes 7 and 8, the antinodes that are 22 nm apart at 180° become one antinode at 30°. Even in the case of modes 3 and 4 that do not merge at 30°, the antinode

Edson P. Bellido, Isobel C. Bicket, Gianluigi A. Botton, McMaster University, Department of Materials Science and Engineering, Hamilton, Canada, e-mail: edsonpazur@gmail.com
Isobel C. Bicket, McMaster University, Canadian Centre for Electron Microscopy, Department, Hamilton, Canada, e-mail: bicketic@mcmaster.ca
***Corresponding author: Gianluigi A. Botton,** Canadian Light Source, Saskatoon, Canada, e-mail: gbotton@mcmaster.ca

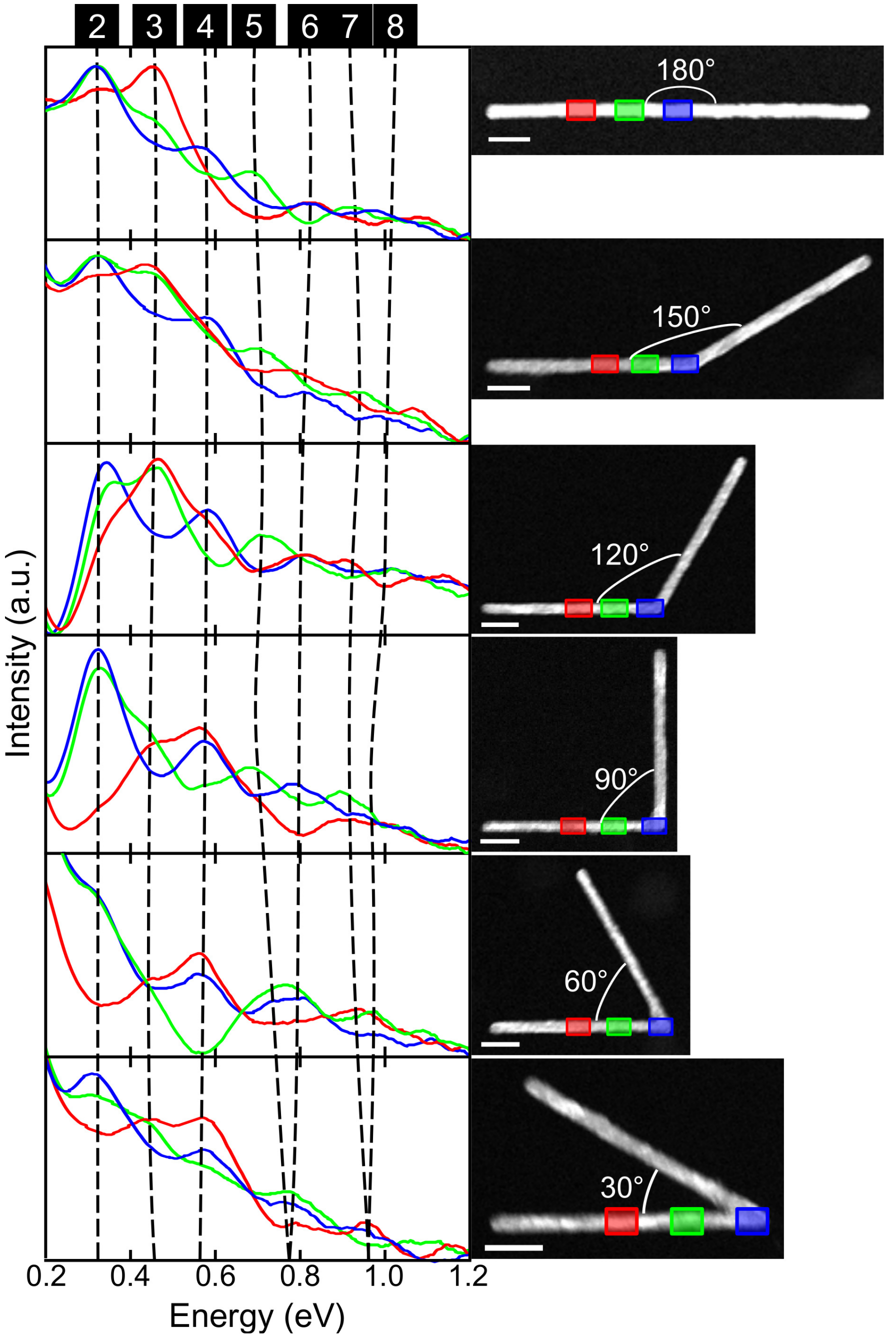

Fig. S 1: Evolution of the 2 μm long NWs' EELS spectra as a function of bending angle (left) acquired at three color-coded locations shown in the annular dark field (ADF) images (right). The scale bar in all ADF images is 200 nm.

distance between the modes is reduced from 36 nm in the 180° NW to only 10 nm in the 30° NW. This is a clear indication of antinode clustering or bunching produced by the presence of a bend in the NWs.

Figures S5, S6, and S7 show the evolution of surface charge and electric field distributions under an asymmetric point electron beam excitation at one tip of the NW. Two examples for a high aspect ratio NW (*i.e.*, the NW in Figure 4a) are shown in Figure 5, at 90° and 30° bend angles. We observe the clear modal distribution of each mode excited, though at higher energies and higher bend angles, the charge antinodes further from the electron beam are less strongly excited for mode 6, indicating that the charges induced in the lower half have difficulty inducing the symmetric charge distribution in the upper half of the bent wire.

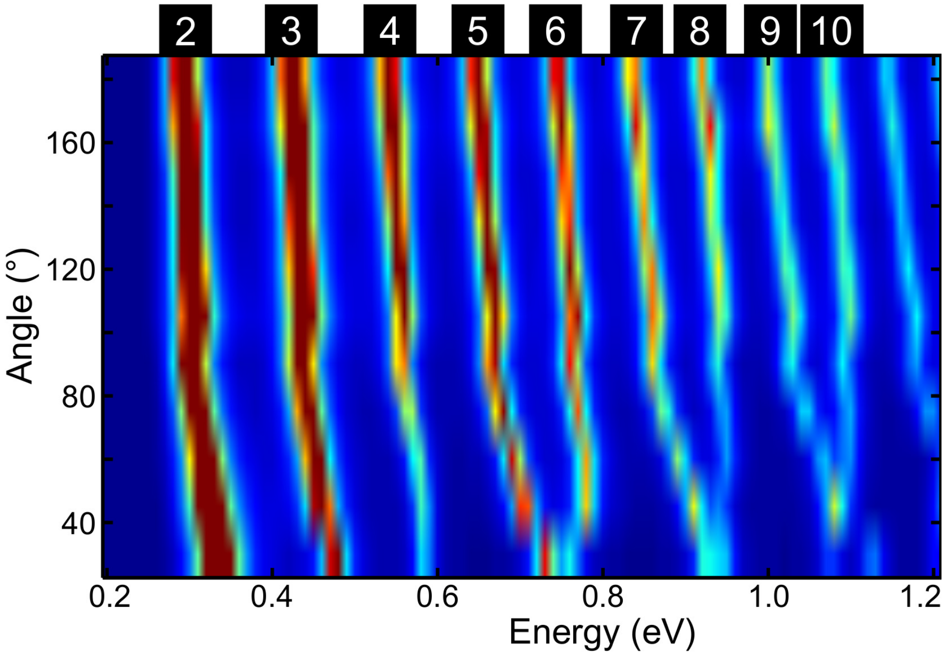

Fig. S 2: Simulated EELS spectra, shown in temperature scale, displaying the change in mode energy as a function of bending angle in 2 μm NWs (28.6 aspect ratio) at $\frac{1}{5}$ scale. The spectra show the energy shifts of the resonant modes which, at small bending angles, cause even and odd modes to intersect in energy.

In lower aspect ratio NWs, we observe the changes in the electron beam-induced modes 3 and 4 as the bend angle is increased in Figure S6, and in modes 5 and 6 in Figure S7. In the high angle bends, we observe the expected patterns, though, as with the high aspect ratio NW, the charges of the even modes (4 and 6) do not propagate well around the bend or couple easily across the gap, so the nodal distribution is not easily observed in the upper half of the NW, far from the electron beam. The opposite is true for the odd modes (3 and 5), where an electron beam near one tip induces strong charges in the opposite half of the NW. It is important to remark that the point electron beam simulations are different from the eigenmode simulations, in which the resonant modes are clearly separated. The point excitation simulations show how the charge density propagates even in non-resonant conditions and can not clearly separate eigenmodes, particularly when these modes are close in energy, because the probability of exciting multiple modes is non-zero.

As the bend angle approaches the angle at which the modes intersect, the odd mode begins to dominate the excited surface charge distribution, particularly near the bend itself. However, we can clearly observe, for example in the 30° bent structure in Figure S6, that the tips of the NWs have opposite charges in the higher energy mode, and same-charges on the lower energy mode, the opposite of the case for the larger bend angles (*e.g.* 90°).

In Figure S8, we observe the eigenmode distribution for similar NWs to those presented in Figure 5 in the main text, completing the set of bending angles from 180° to 0°. The effects of bending on the surface charges are not yet obvious at low bending angles, particularly for the high aspect ratio NWs. At 0° bending angle, the NWs are represented as half-length NWs with double the width. The identification of the evolution of Mode 3 at 0° is not clear, as the complex charge distribution with two opposing antinodes coming into contact may become a much higher order mode not identified in these simulations. Mode 4, however, evolves into the second eigenmode for the half-length NW as two same-charge antinodes come into contact and merge when the bending angle is reduced to the limit.

The relation between edge modes and the modes in a nanowire can be analyzed by topologically transforming a NW into a nanosquare, which supports edge modes. Similar to the case of NW modes, edge modes can be identified by the number of nodes n along the edge, and are denoted here as E_n. Figure S9A

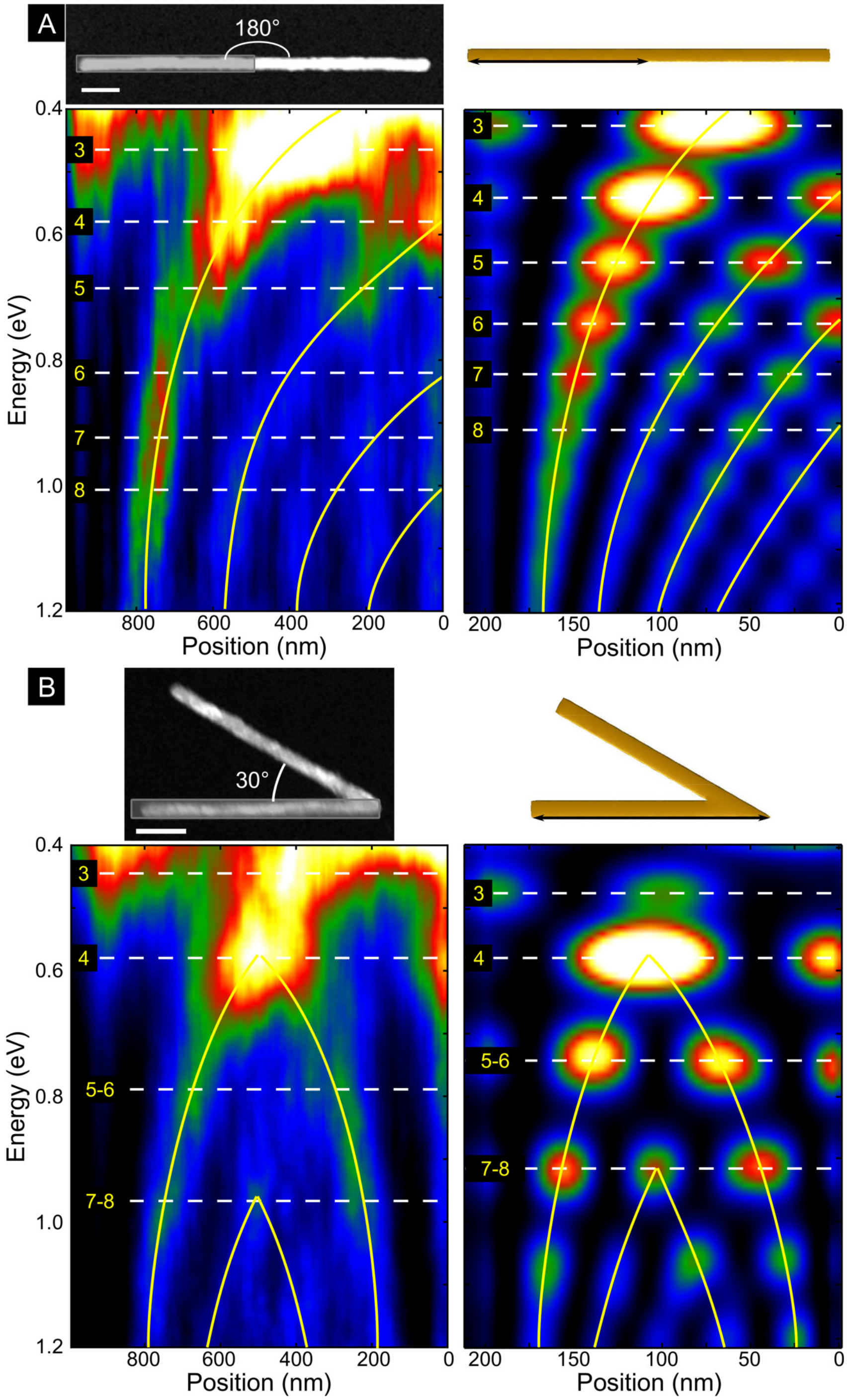

Fig. S 3: Experimental and simulated 2D EELS intensity profile of a straight nanowire (A) and a $30°$ bend nanowire (B) showing how the nodal distribution of the edge modes changes due to the presence of a kink. The simulated profile is at $\frac{1}{5}$ scale. The profiles were acquired in the gray regions shown in the ADF images and along the arrows in the simulation. The scale bars are 200 nm.

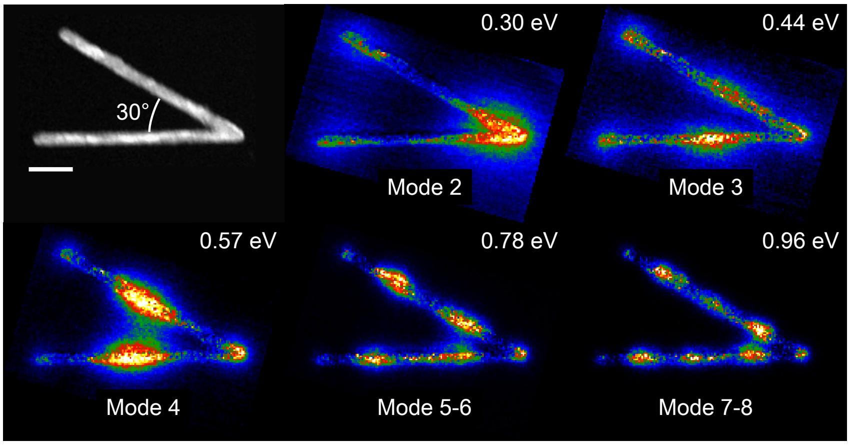

Fig. S 4: EELS maps of the 2 μm long and 30° bend nanowire showing the symmetry of the nodal distribution across both halfs of the NW. The maps are integrated over a 40 meV window and the scale bar is 200 nm.

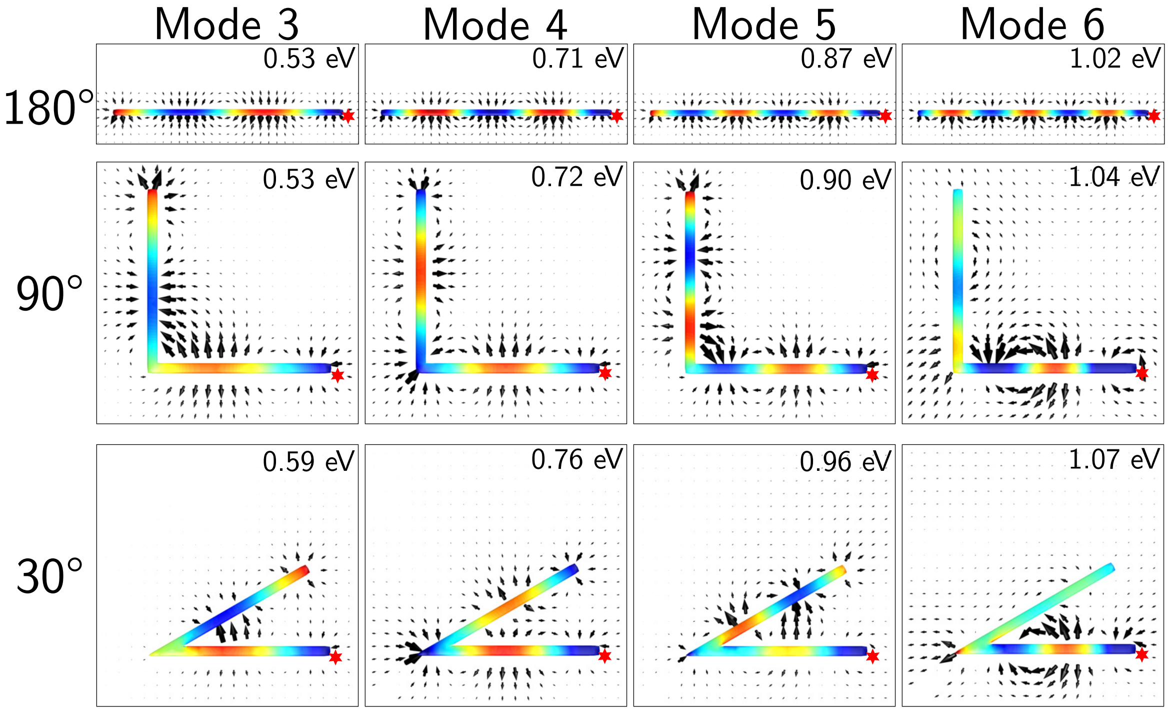

Fig. S 5: Simulated surface charge distributions of the NWs (modes 3-6) presented in Figure 4a, unbent (180°), and bent to angles of 90° and 30°, under excitation by an electron beam at the location indicated by the red star. The NWs are 850 nm long and 20 nm wide, with an aspect ratio of 42.5.

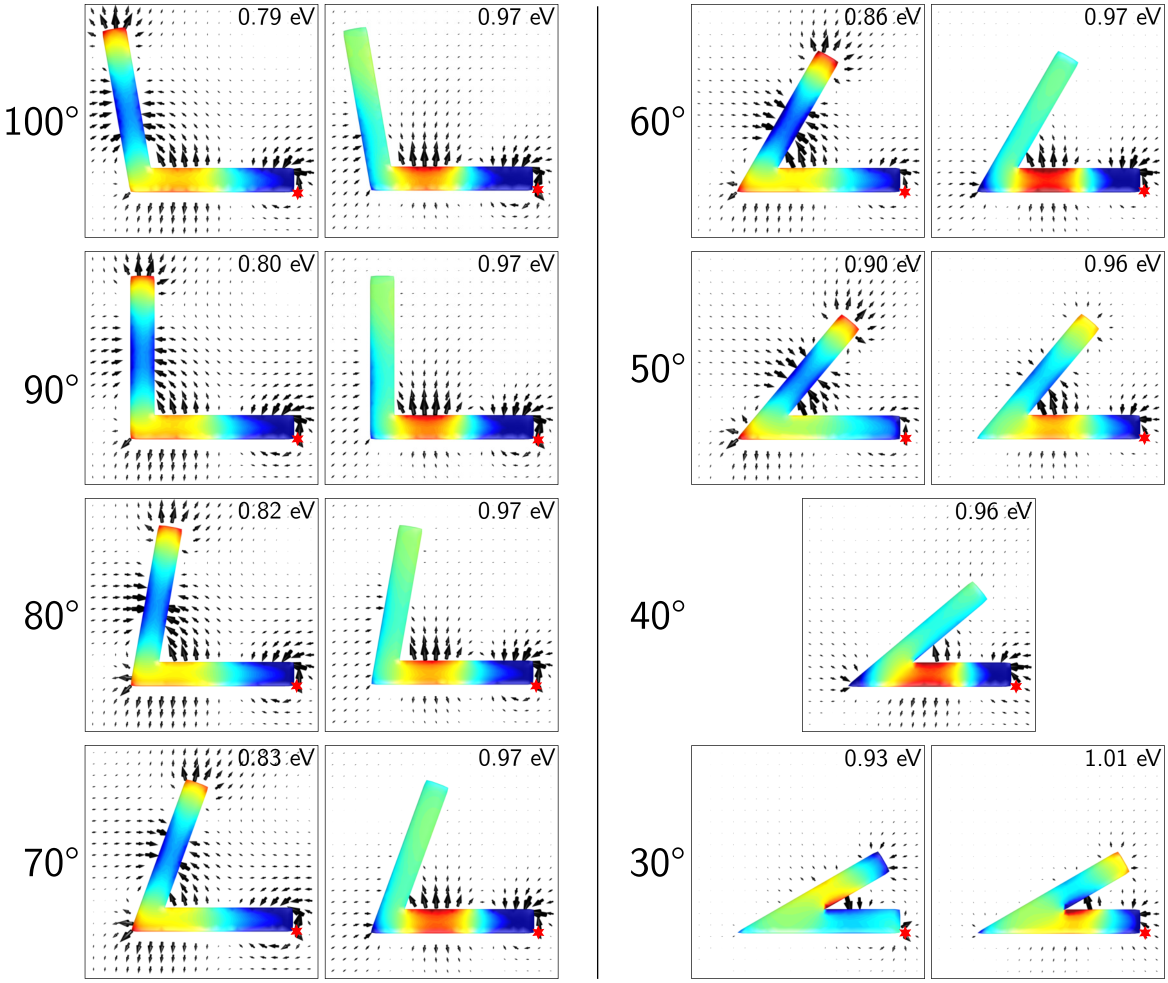

Fig. S 6: Simulated surface charge distributions of modes 3 and 4 in the NWs presented in Figure 4b, as a function of bend angle, under excitation by an electron beam at the location indicated by the red star. The NWs are 850 nm long and 60 nm wide, with an aspect ratio of 14.2

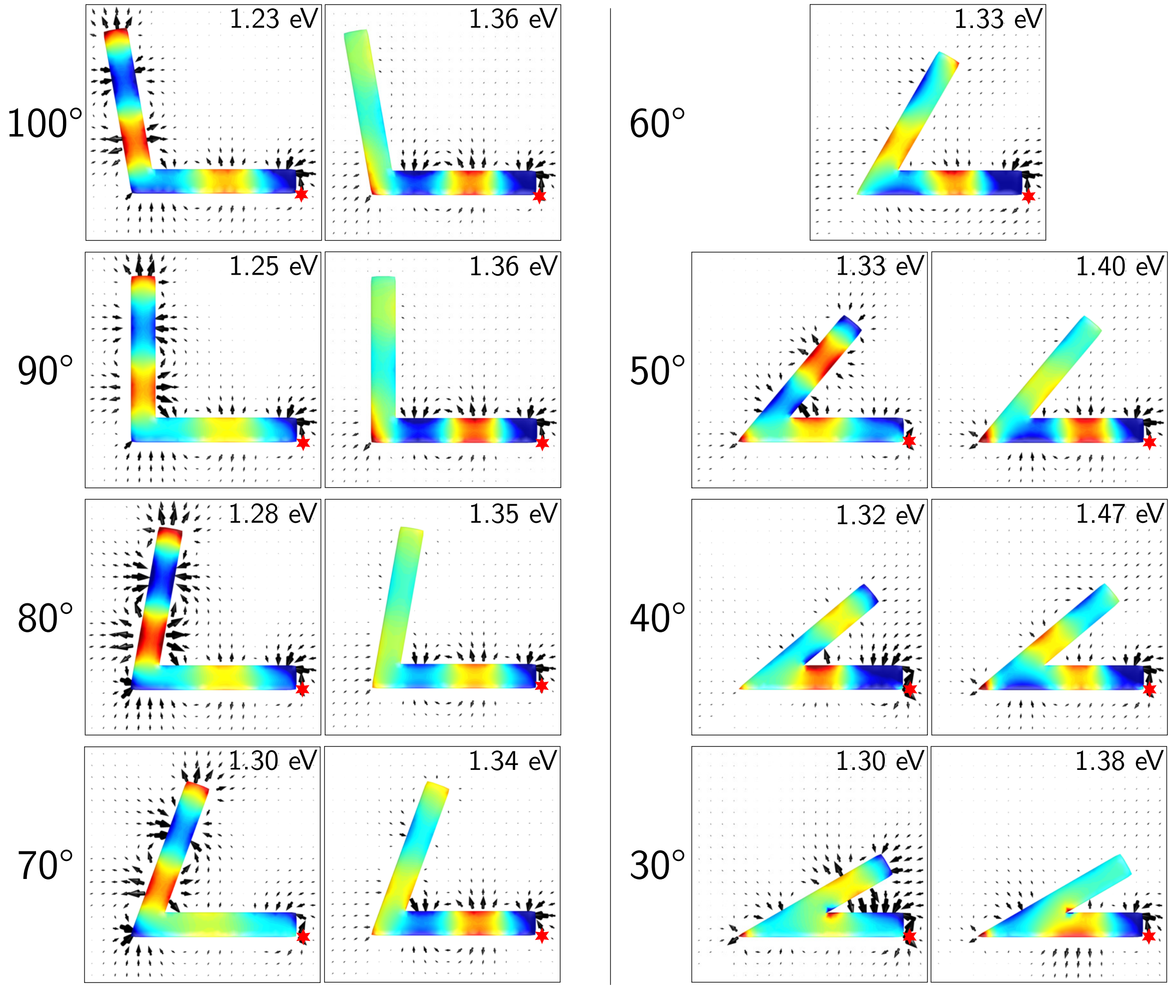

Fig. S 7: Simulated surface charge distributions of modes 5 and 6 in the NWs presented in Figure 4b, as a function of bend angle, under excitation by an electron beam at the location indicated by the red star. The NWs are 850 nm long and 60 nm wide, with an aspect ratio of 14.2.

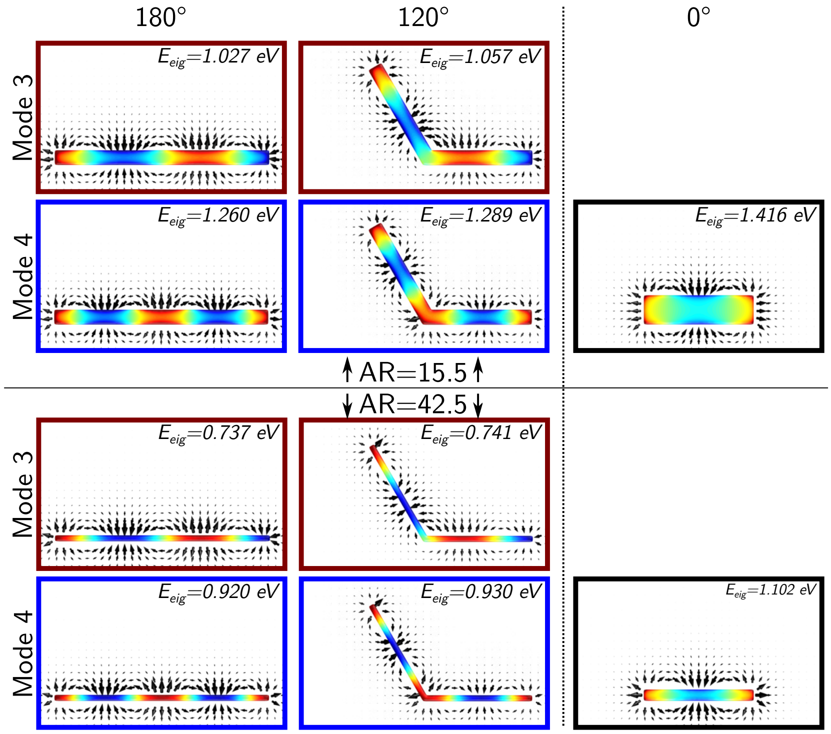

Fig. S 8: Evolution of eigenmodes 3 and 4 for nanowires with aspect ratios (AR) of 15.5 (top rows) and 42.5 (bottom rows) for bending angles of 180°, 120°, and 0°, which is effectively represented as a nanowire of half the length and double the width.

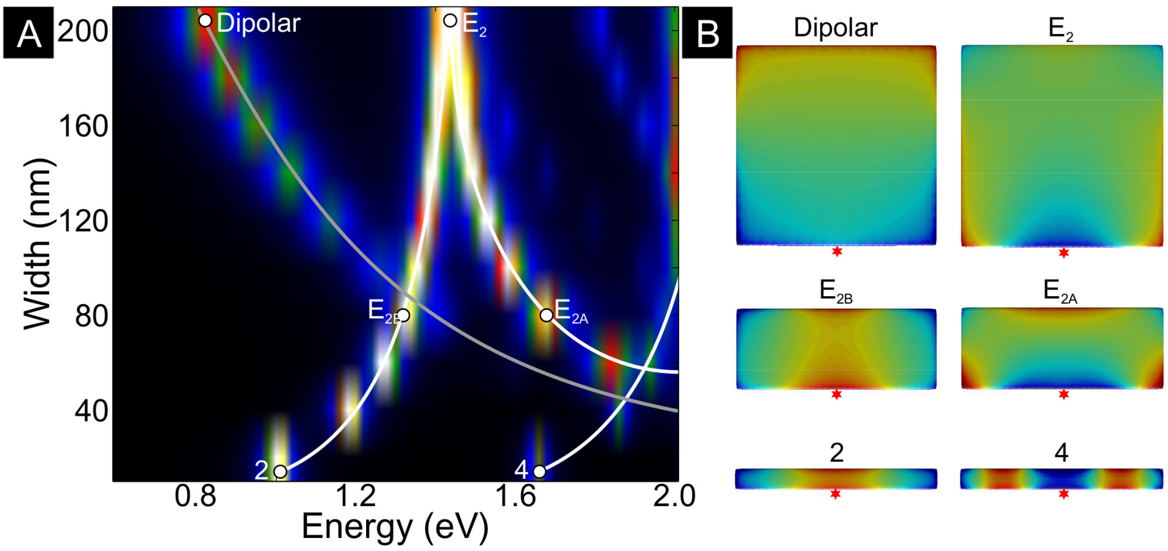

Fig. S 9: Simulated EELS distributions shown in a temperature scale as the width of a nanowire changes from 20 nm to 200 nm when it transforms into a nanosquare (A). The spectra are calculated at the center of one edge of the nanostructures, as indicated by red stars in panel (B). Charge distribution of the edge modes (B), calculated at the energies indicated by the white dots in (A). The Figure shows the relationship between the NW modes and the edge modes of a nanosquare. E_{2B} refers to the second bonding mode while E_{2A} refers to the second antibonding mode.

shows simulated EELS spectra calculated at the center of one edge of a NW, indicated by the red stars in Figure S9B, as we transform it into a nanosquare. We observe that, as the width of the NW increases,

modes 2 and 4 of the NW shift to higher energies. If the width of the NW keeps increasing, we observe that another mode appears in the analyzed range of energies. This mode can be identified as antibonding edge mode E_{2A}, shown in the charge distribution in Figure S9B. The charge distribution of mode 2 in the NW/nanorectangle with a width of 80 nm shows that the antinodes along the length of the NW split into two sets of symmetric antinodes at each side of the NW, characteristic of the bonding edge mode E_{2B}. Therefore, we demonstrate here that mode 2 of a NW is none other than edge mode E_{2B}. As we continue increasing the width of the nanorectangle, transforming it into a nanosquare, we observe that modes E_{2B} and E_{2A} merge into mode E_2. This behavior is typical of the coupling of edge modes within a planar nanostructure, in which, as the distance between edges shortens, the interaction between edge modes is enhanced, resulting in the formation of a bonding and an antibonding mode, as described by hybridization theory. We also notice the presence of a dipolar mode, shown in the charge distribution in Figure S9B. This dipolar mode evolves from the transverse mode found in NWs as the width of the NW increases. These results show that the modes in a planar NW are actually the bonding edge modes formed by the strong coupling of the modes of its edges. This explains the dependence of the resonant energy of the modes on the aspect ratio of the NW. The larger the aspect ratio, the lower the resonant energy of the modes. Alternatively, the larger the aspect ratio of the NW, the stronger the coupling between edges and the larger the red shift of the bonding edge modes.
